# Anti-Proliferative Activity of A Hydrophilic Extract of Manna from *Fraxinus angustifolia* Vahl through Mitochondrial Pathway-Mediated Apoptosis and Cell Cycle Arrest in Human Colon Cancer Cells

**DOI:** 10.3390/molecules25215055

**Published:** 2020-10-30

**Authors:** Ignazio Restivo, Luisa Tesoriere, Anna Frazzitta, Maria Antonia Livrea, Alessandro Attanzio, Mario Allegra

**Affiliations:** Dipartimento di Scienze e Tecnologie Biologiche, Chimiche e Farmaceutiche, Università di Palermo, 90123 Palermo, Italy; ignazio.restivo@unipa.it (I.R.); luisa.tesoriere@unipa.it (L.T.); anna.frazzitta@unipa.it (A.F.); maria.livrea@unipa.it (M.A.L.)

**Keywords:** *Fraxinus angustifolia*, manna, apoptosis, anticancer, colon cancer cells

## Abstract

Manna is produced from the spontaneous solidification of the sap of some *Fraxinus* species, and, owing its content in mannitol, is used in medicine as a mild laxative. Manna is also a rich source of characteristic bio-phenols with reducing, antioxidant and anti-inflammatory properties. This study assesses the activity of a hydrophilic extract of manna (HME) on cellular and molecular events in human colon-rectal cancer cells. HME showed a time- and concentration-dependent anti-proliferative activity, measured by MTT assay, in all the cell lines examined, namely Caco-2, HCT-116 and HT-29. The amounts of HME that caused 50% of cell death after a 24 h treatment were 8.51 ± 0.77, 10.73 ± 1.22 and 28.92 ± 1.99 mg manna equivalents/mL, respectively; no toxicity was observed in normally differentiated Caco-2 intestinal cells. Hydroxytyrosol, a component of HME known for its cytotoxic effects on colon cancer cells, was ineffective, at least at the concentration occurring in the extract. Through flow-cytometric techniques and Western blot analysis, we show that HME treatment causes apoptosis, assessed by phosphatidylserine exposure, as well as a loss of mitochondrial membrane potential, an intracellular formation of reactive oxygen species (ROS), increases in the levels of cleaved PARP-1, caspase 3 and Bax, and a decrease in Bcl-2 expression. Moreover, HME interferes with cell cycle progression, with a block at the G1/S transition. In conclusion, the phytocomplex extracted from manna exerts an anti-proliferative activity on human colon cancer cells through the activation of mitochondrial pathway-mediated apoptosis and cell cycle arrest. Our data may suggest that manna could have the potential to exert chemo-preventive effects for the intestine.

## 1. Introduction

Colorectal cancer, one of the main causes of morbidity and mortality worldwide, is a multi-stage disorder due to genetic and epigenetic alterations the accumulation of which is influenced by the micro-environment throughout life [1]. This understanding explains the relentless efforts being made in the study of foods and natural compounds that can be harmful or, conversely, act positively to modulate the intestinal cell environment, and ultimately prevent or reverse the aberrations that lead to uncontrolled cell division. Mounting evidence indicates that dietary phytochemicals, particularly polyphenols, can play critical roles in modulating the signaling pathways governing intestinal cell well-being, and exert potential chemo-preventive effects on the intestine. A combination of antioxidant, anti-proliferative, anti-inflammatory and pro-apoptotic effects, along with the induction of cell cycle arrest, is considered to be the basis of their action [2,3].

So-called “manna” is the product of the sap of some species of Sicilian *Fraxinus*, spilled and gathered according to a traditional practice [4]. Manna has a long history in folk medicine and is a consolidated natural remedy against constipation, due to its main component mannitol, an osmotically active cell-compatible polyol [5]. We have recently reported on the extraction and characterization of a polyphenol phytocomplex from manna (hydrophilic manna extract, HME), with reducing and antioxidant activity in several chemical and biological in vitro systems [6]. Moreover, HME prevented redox imbalance and inhibited the inflammatory response in differentiated colon cells activated by IL-1β. These data prompted us to investigate further the phytocomplex of manna, to extend knowledge on the bioactivity of this product and possibly reveal its novel functional aspects.

In this work, we evaluate for the first time the effects of HME against the proliferation of human colorectal cancer HCT-116, Caco-2 and HT-29 cells, which are three of the fastest growing cell lines used as colon cancer models [7]. We present evidence that HME causes cell cycle arrest and induces the apoptosis of cancer cells via the mitochondrial pathway, mediated by the increase of intracellular ROS. Our data suggest that, beyond its well-known effects against constipation, manna may have chemo-preventive potential for the intestine.

## 2. Results

### 2.1. HME Inhibits the Proliferation of Human Intestinal Cancer Cells

**Figure 1:**
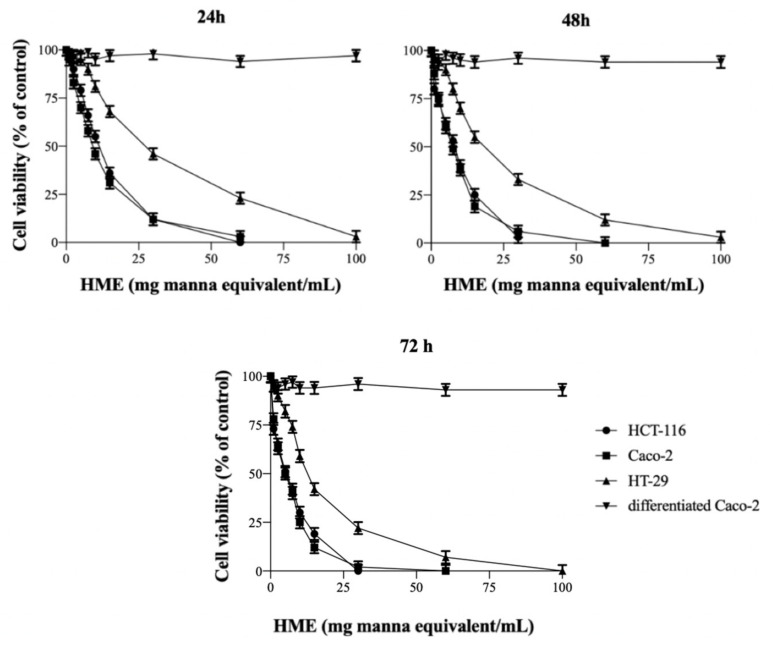
Inhibitory effect of HME on the growth of colon cancer cells. Cell monolayers were incubated for 24–72 h with HME. Cell viability was assessed by MTT test as reported in the Methods. Results are indicated as the percentage of viable cells with respect to untreated controls. Values are the mean ± SD of three separate experiments carried out in triplicate.

The cytotoxicity of HME, i.e., its ability to inhibit the proliferation of HCT-116, Caco-2 and HT-29 cells, has been assessed by the MTT assay, after treatment with increasing amounts of the extract for 24–72 h (Figure 1). The IC_50_, that is, the amount of HME causing a 50% inhibition of cell vitality, was calculated as 10.73 ± 1.22, 8.51 ± 0.77 and 28.92 ± 1.99 mg manna equivalents/mL (*n* = 4) for HCT-116, Caco-2 and HT-29 cells, respectively, at 24 h, and this decreased with the duration of treatment between 24 and 72 h (Table 1). The data indicate the cytotoxic activity of HME and the anti-proliferative effect of the manna phytocomplex in all cell systems. Conversely, the viability of normally differentiated Caco-2 cells did not change when cells were treated with HME, even after long incubation times, indicating the selective toxicity of HME towards colon cancer cells (Figure 1).

Interestingly, when HCT-116, Caco-2 and HT-29 cells were incubated for 24 h with cisplatin as a positive control, the resulting IC_50_ values were 40.1 ± 3.2, 134.6 ± 6.3 and 169.1 ± 4.2 µM, respectively.

Among the phytochemical components of HME, hydroxytyrosol (HT) at about 15 µg/g has been reported to be involved in the inhibition of multiple stages of colon carcinogenesis in several studies [6,8,9]. The effect of pure HT on the growth of colon tumor cells has been assessed at 2.5, 5.0 and 10 µM, consistently with its concentrations in the HME of 30, 60 and 120 mg manna equivalents/mL, to investigate to what extent this component would contribute to the anti-proliferative activity of the extract. The viabilities of the HCT-116, Caco-2 and HT-29 cells were not significantly different (*p* > 0.05) from those of the untreated cells after the 24 to 72 h treatments, at any HT concentration (not shown), which apparently ruled out the contribution of HT to the anti-proliferative activity of our manna extract under these conditions.

Decrease of cell viability could be due to cell growth inhibition and/or apoptosis induction. HCT-116 cells were selected to investigate the effect of HME on apoptosis and cell cycle progression.

### 2.2. HME Induces Mitochondrial-Mediated Apoptosis in HCT-116 Cancer Cell Line

**Figure 2:**
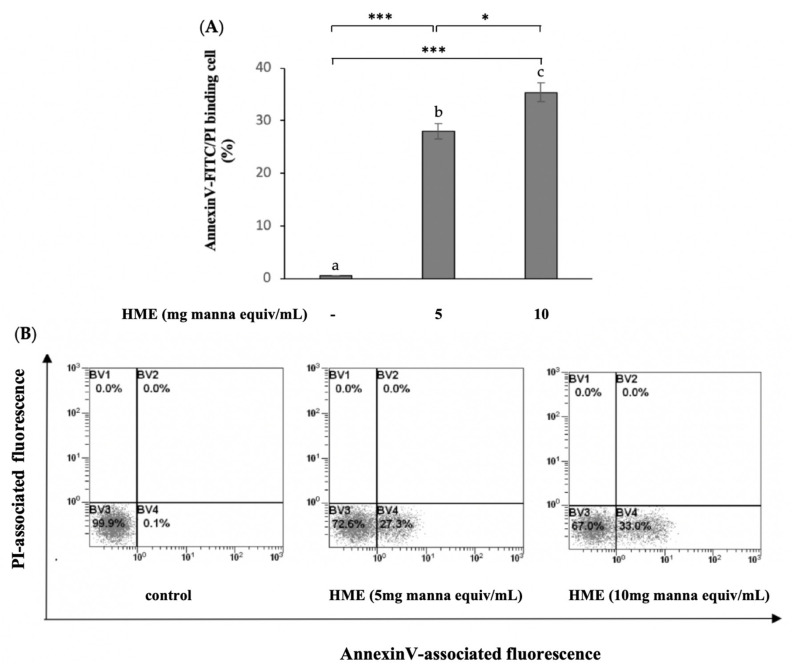
Apoptosis induced by HME on HCT-116 cells. Cell were treated for 24 h as reported in Methods. Percentages of AnnexinV/PI double stained-HCT-116 cells were determined by a flow cytometer and compared to untreated cells (control). (**A**) Mean values ± SD of three separate experiments in triplicate. Means with different letters are significantly different (one-way Anova associated with Tukey’s post hoc test) with * *p* < 0.05, *** *p* < 0.001. (**B**) Representative images: BV3, viable cells (AnnexinV−/PI−); BV4, cells in early apoptosis (AnnexinV+/PI−); BV2, cells in tardive apoptosis (AnnexinV+/PI+); BV1, necrotic cells (AnnexinV−/PI+).

Apoptosis induction is considered an important goal in a preventive approach against cancer, by the conversion of a normal cell to a malignant one. The PS-exposure of HCT-116 cells treated for 24 h with the HME from 5 and 10 mg manna equiv/mL was measured by flow cytometry using Annexin V-FITC/PI double staining to assess the fraction of apoptotic cells. In comparison with untreated cells, the percent of apoptotic cells significantly increased in the HME-treated HCT-116 cells; the higher the amount of HME the greater the number of AnnexinV-FITC fluorescent cells (*p* < 0.05, Figure 2).

**Figure 3:**
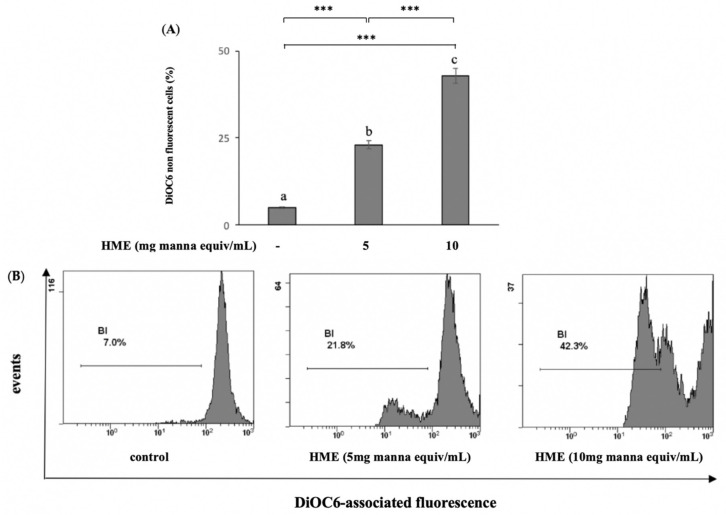
Depolarization of mitochondrial membrane potential induced by HME on HCT-116 cells. Cells were treated for 24 h as reported in Methods. Then the percentages of DiOC6 stained-HeLa cells were determined by a flow cytometer and compared to untreated cells (control). (**A**) Mean values ± SD of three separate experiments in triplicate. Means with different letters are significantly different (one-way Anova associated with Tukey’s post hoc test) with *** *p* < 0.001. (**B**) Representative images.

The mitochondrial membrane potential (MMP) value is a key indicator of mitochondrial activity. MMP collapse is an early marker of mitochondrial dysfunction associated with cell apoptosis. The measurement of MMP was performed using the fluorescent, mitochondria-specific and voltage-dependent dye DiOC6. Treatment for 24 h of HCT-116 cells with the HME from 5 and 10 mg manna equiv/mL resulted in reductions in the fluorescence intensity of the probe of about 23.2 ± 1.9% and 43.4 ± 2.3%, respectively, compared to the untreated cells (Figure 3), indicating that the apoptotic activity of manna was mediated by mitochondria.

**Figure 4:**
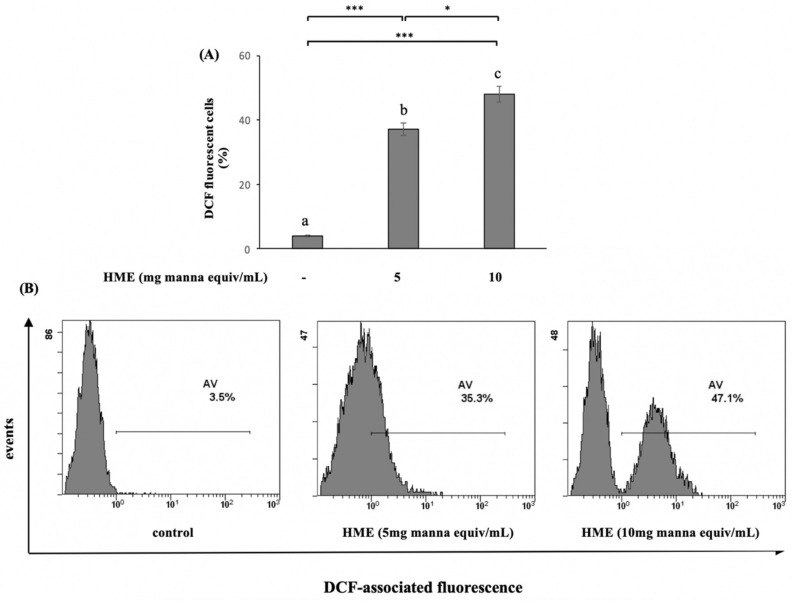
ROS production induced by HME on HCT-116 cells. ROS levels were cytofluorimetrically evaluated after 24 h treatment by staining of the cells with DCFDA as reported in Methods. Data are compared to untreated cells (control). (**A**) Mean values ± SD of three separate experiments in triplicate. Means with different letters are significantly different (one-way Anova associated with Tukey’s post hoc test) with * *p* < 0.05; *** *p* < 0.001. (**B**) Representative images.

Since the generation of intracellular ROS may be related to mitochondrial dysfunction and the induction of apoptosis in various cell types, we explored whether HME stimulated ROS generation. As illustrated in Figure 4, the generation of ROS, cytofluorometrically detected by the fluorescent dye DCFH-DA, dramatically increased in the HME-treated HCT-116 cells compared with the untreated ones, with a clear concentration-dependent effect.

**Figure 5:**
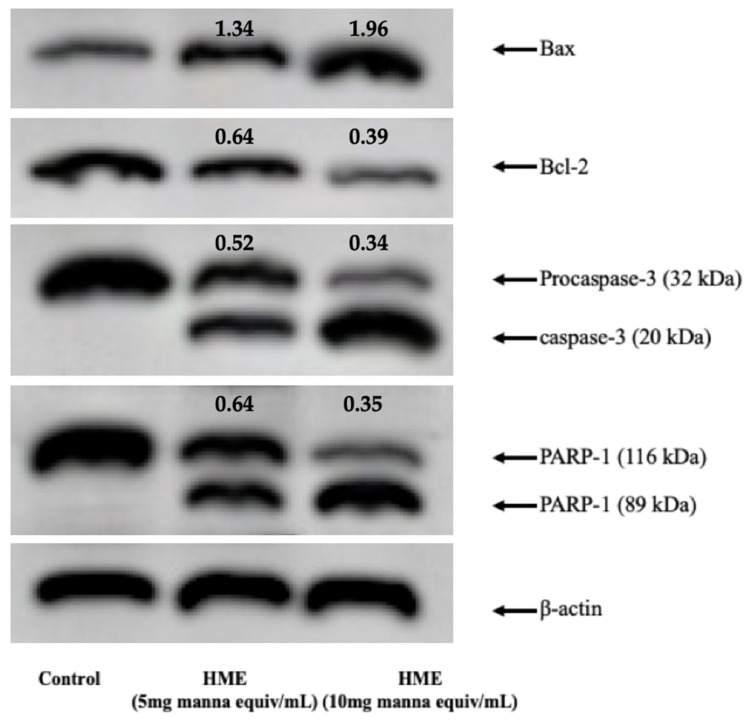
Effect of HME on the levels of apoptotic proteins on HCT-116 cells. Cells were treated for 24 h as described in Methods. Representative images of immunoblotting analysis of Bax, Bcl-2, procaspase-3 and PARP-1 levels of three separate experiments with comparable results; values were normalized on β-actin levels. Numbers above the bands represent the densitometric ratio with the control.

Western blotting analysis was performed to investigate proteins associated with mitochondria-mediated apoptosis in HME-treated HCT-116 cells, including Bax, Bcl-2, caspase 3 and poly ADP ribose polymerase (PARP). Bax and Bcl-2 are the two main factors regulating cell survival vs. death [10]. In addition, the cleavage of PARP-1 by caspases is considered to be a hallmark of apoptosis [11]. Caspase-3, in particular, cleaves PARP-1 to yield an 89-kD PARP-1 fragment [12]. As exhibited in Figure 5, in comparison with untreated cells (control), treatment with HME (5 and 10 mg manna equiv/mL) remarkably increased the protein expression levels of Bax, and decreased Bcl-2 expression, in a concentration-dependent manner. Moreover, the HME treatment decreased the level of caspase-3 and PARP-1 proteins, with accumulation of the relevant cleaved forms (Figure 5).

### 2.3. HME Induces Cell Cycle Arrest in HCT-116 Cancer Cell Line

**Figure 6:**
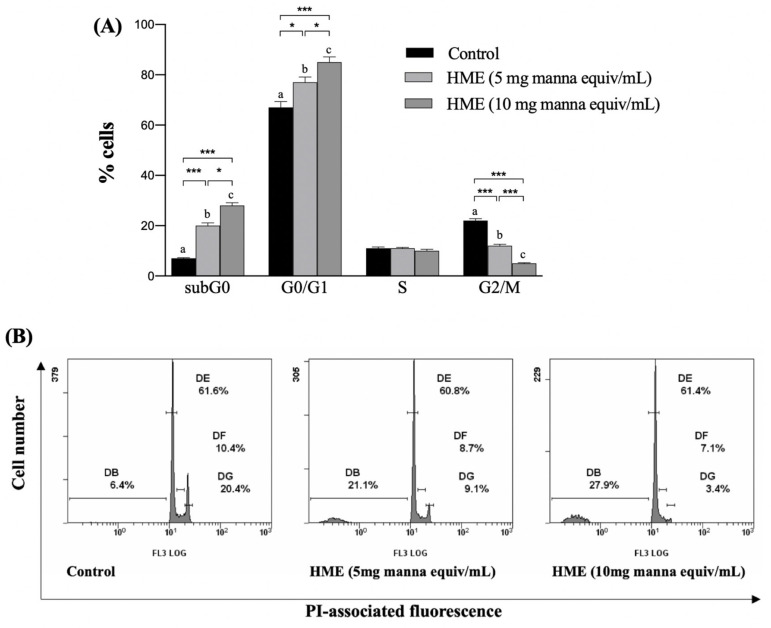
Effect of HME on the cell cycle distribution of HCT-116 cells. Cell were treated for 24 h as reported in Methods. Then, samples were submitted to flow cytometry analysis after propidium iodide (PI) staining and cell distribution compared to untreated cells (control). (**A**) Mean values ± SD of three separate experiments in triplicate. Within each group, means with different letters are significantly different (one-way Anova associated with Tukey’s post hoc test) with * *p* < 0.05; *** *p* < 0.001. (**B**) Representative images.

Cellular proliferation is regulated by the cell cycle progression, therefore an uncontrolled cell cycle is a hallmark for cancer and a target for anti-cancer treatments. HCT-116 cells were treated with the HME from 5 or 10 mg manna for 24 h, followed by FACS analysis of the PI-labeled cell nuclei. In comparison with untreated cells (control), a concentration-dependent increase in cells in the G0/G1 and a decrease in the G2/M phase were observed (Figure 6), indicating a blocking of the cell cycle at the G1/S transition. The increase in the fluorescence of cells in the sub G0/G1 phase is indicative of nuclear DNA fragmentation, and supports the cytotoxic activity of HME (Figure 6).

## 3. Discussion

A highly regulated balance between apoptosis and proliferation plays a physiological function to maintain a normal tissue homeostasis; excess apoptosis can contribute to degenerative age-related disorders, whereas inhibition can induce abnormal cell proliferation [13,14,15]. In line with the highly effective physiologic apoptosis of the colon epithelial cells, colon–rectal cancer has appeared as particularly associated with a progressive inhibition of this process [16,17]. Plant-derived natural agents that induce apoptosis, either single compounds or whole extracts, have appeared as important targets for colon cancer research [18]. We have recently isolated and characterized a highly reducing polyphenol-rich phytocomplex from the manna of *Fraxinus angustifolia* Vahl, and demonstrated that it has a remarkable activity in preserving the redox balance and preventing the release of inflammatory cytokines in human colon cells activated by IL-1β [6]. This work shows that this phytocomplex inhibits the proliferation and induces the apoptosis of human colon cancer cells, indicating that besides contrasting the effects of noxious stimuli in normal cells [6], it can interfere with already up-regulated cell signals that support proliferation in the neoplastic ones.

Investigations of the cytotoxic effect in HCT-116 cells have demonstrated that HME induces apoptosis with reduction in mitochondrial membrane potential and the production of reactive oxygen species. In addition, the expression level of proteins functional to apoptosis has indicated that the signals triggered by the manna phytocomplex involve the mitochondria-mediated apoptotic pathway [19].

The key event in mammalian cell replication is the entry into and progression of quiescent G0 cells through the cell cycle. This highly regulated signaling pathway comprises the G1, S, G2 and M phases, with the G1/S transition being the restriction point of the cycle that represents the rate-limiting step in the cell cycle’s progression [20]. A deregulated cell cycle is a hallmark for cancer. We observed that HME interfered with this process in the HCT-116 tumor cells, with a crucial blockage at the G1/S transition and a reduction in cells in the G2/M phase, which supported its anti-proliferative activity. Apoptosis and cell proliferation are linked by stimuli that affect both processes [21]. In accordance, the increase in the sub G0/G1 phase observed in HME-treated vs. control cells confirmed that the treated cells had undergone apoptosis.

Antioxidants/redox-active molecules may interact with each other, so natural mixtures containing redox-active compounds may affect cell environments in a far different way from any single compound. Colon carcinogenesis [22] involves multiple phases with signaling pathways that may be sensitive to different agents, and that may be better targeted by a combination of bioactive compounds in a natural extract, rather than a single purified component [23,24]. Hydroxytyrosol is a major polyphenol of the manna extract [6], known to inhibit carcinogenesis in a remarkable number of tumor cell systems, including colorectal cells [25,26]. However, we observed that HT, at concentrations consistent with the amount in the whole extract, did not affect the growth of the colon tumor cells, suggesting that the anti-proliferative effectiveness of HME has to be ascribed to other component(s), or rather to the combined action of its phytochemicals. Some phenolic compounds of manna, including HT, tyrosol and secoiridoids [6], are common to olive oil [27,28], which is in line with the chemotaxonomic closeness between Fraxinus and Olea genera. In accordance with our findings, Fabiani et al. [29] observed the high anti-proliferative capacity of an olive oil extract accounting for 0.1 µM HT, whereas pure HT was effective only at 100 µM under comparable conditions [30]. Other research showed that low amounts of HT and tyrosol, ineffective per se, synergistically suppressed the growth of HT-29 colon cancer cells when combined [8]. In this context, interactions with fraxetin, a unique hydrocoumarin of the *Fraxinus* species [31] that occurs in HME [6] with reported anti-proliferative activity in various cancer cell lines [32,33], may deserve an investigation as regards colon cancer cells.

Chemo-prevention is now considered an essential strategy to fight against cancer. Indeed, multiple molecular events inducing the early phases and development of tumorigenesis may be modulated over time, to prevent the onset of cancerous lesions [34,35,36]. Among other events, inflammation plays a critical role [37,38]. Using physiological mechanisms, apoptosis included [39], chemo-preventive agents, including plant-derived products and their components [36,37], can block the initiation, reverse the promotion, and halt or retard the progression of precancerous cells into malignant ones. In this context, manna is promising. We here show that manna contains a pool of phytochemicals that exert anti-proliferative activity and induce apoptosis in human colon cancer cells. Moreover, due to the crucial role of inflammation in the induction and progression of colorectal carcinoma [40,41,42], it is meaningful that the manna extract also possesses a remarkable anti-inflammatory activity in colon cells [6].

Remarkably, this work shows that the phytocomplex present in the HME exerts its antiproliferative effects at nutritionally-relevant concentrations. Indeed, considering the IC_50_ measured in the colon cell lines at 24 h, and considering an intestinal volume of 600 mL, the amounts of HME exerting anti-proliferative activity are compatible with the assumption of 6 g to 18 g manna [6]. Of interest, the approximately laxative threshold of mannitol in humans has been measured as 10 g to 20 g of oral administration [43]. Finally, and noteworthy, the antiproliferative effects of HME are selectively directed onto colon cancer cells, as normally differentiated Caco-2 cells do not suffer from cytotoxicity even when they are treated with HME for considerable incubation times.

In conclusion, our present and previous observations on the bioactivity of the manna phytocomplex suggest that dietary manna could provide chemo-preventive protection to colon cells, the first target of orally ingested compounds. Further research into in vivo models is warranted to evaluate long-term safety and efficacy.

## 4. Materials and Methods

### 4.1. Hydro-Alcoholic Extract of Manna (HME)

HME was prepared in August 2018 and characterized by HPLC-DAD as reported by Attanzio et al. [6]. Briefly, aliquots of 30% (*w*/*v*) aqueous manna solution were extracted with two volumes of methanol at room temperature for 1 h. Then the hydro-alcoholic extract was placed at 4 °C overnight and crystallized sugar was removed by centrifugation at 3000 g for 10 min. The supernatants were withdrawn and submitted to rotary evaporation at 35 °C. Water residual from the solution was then eliminated by cryodessication. Dried samples of HME were stored at −80 °C and used within two months to undertake the current study. Before use, dried HME was resuspended in suitable volumes of cell culture medium and filtered through 0.2 μm Millipore filters (Milan, Italy).

### 4.2. Cell Culture and Treatment

The cancer cell lines used for the experiments (Caco-2, HCT-116, HT-29) were obtained from the American Type Culture Collection (Rockville, MD, USA). All of them were epithelial adherent cells of human colorectal adenocarcinoma and were cultured in Dulbecco’s modified Eagle medium (DMEM; Gibco Life Technologies, Grand Island, NY, USA) supplemented with 10% fetal bovine serum (Gibco Life Technologies), 2 mM l-glutamine, 1% non-essential amino acids, 10 mM HEPES, 50 units/mL penicillin, 50 µg/mL streptomycin, and 100 µg/mL gentamicin. All the cells were maintained in log phase by seeding twice a week at a density of 5 × 10^4^ cells/cm^2^ in humidified 5% CO_2_ atmosphere.

### 4.3. Viability Assay

The cytotoxic activity of the HME against the tumor cell lines was determined by the MTT colorimetric assay based on the reduction of 3-(4,5-dimethyl-2-thiazolyl)bromide-2,5-diphenyl-2-*H*-tetrazolium to purple formazan by the mitochondrial dehydrogenases of living cells. This method is commonly used for inhibition assays of cellular proliferation. Cells were considered suitable for the experiment at passage that did not exceeded the number 20. For the experiments, the cells were seeded into 96-well culture plates (Corning Costar Inc., Corning, NY, USA) at a density of 2.0 × 10^4^ cells/cm^2^, left to incubate overnight to allow adhesion, before treatment with HME or vehicle (control) for 24 to 72 h. In selected experiments, Caco-2 cells were treated 15 days after confluence, at which time the cells are differentiated in normal intestinal-like cells [44]. After treatment, the medium was carefully removed, the cells were washed and 50 μL FBS-free medium containing 5 mg/mL MTT was added. The medium was discarded after a 2 h incubation at 37 °C, and the formazan blue formed was dissolved in DMSO. The absorbance at 565 nm of MTT-formazan was measured in a microplate reader (Bio-Rad, Hercules, CA, USA), and the value of the control cells was taken as 100% of viability.

### 4.4. Flow Cytometry

#### 4.4.1. Measurement of Phosphatidylserine Exposure

The flow cytometer cell sorter (FACS) determination of the externalization of phosphatidylserine (PS) to the cell surface was performed by the double staining of cells with Annexin V/propidium iodide (PI), in accordance with the Annexin V apoptosis detection kit FITC (Cat. No. 88–8005, eBiosciences Inc., San Diego, CA, USA). Tumor cells were seeded in triplicate in 24-well culture plates at a density of 5.0 × 10^4^ cells/cm^2^. After an overnight incubation, the cells were washed with fresh medium and incubated with HME or vehicle alone (control cells) in DMEM for 24 h. Then the cells were harvested by trypsinization and adjusted at 1.0 × 10^6^ cells/mL with combining buffer, following the manufacturer’s instructions. Cell suspension (100 µL) was added to a new tube, and incubated with 5 µL Annexin V and 10 µl of 20 mg/mL PI solution at room temperature, in the dark, for 15 min. Samples of at least 1 × 10^4^ cells were subjected to FACS analysis by Epics XL™ flow cytometer with Expo32 software (Beckman Coulter, Fullerton, CA, USA) using an appropriate two-dimensional gating method.

#### 4.4.2. Measurement of MMP

MMP was assayed by flow cytofluorometry, using the cationic lipophilic dye 3,3′-dihexyloxacarbocyanine iodide (DiOC6) (Molecular Probes, Inc., Life Technologies Italia, Monza, Italy) that accumulates in the mitochondrial matrix. Loss of MMP is indicated by a reduction in the DiOC6-induced fluorescence intensity. Cells were incubated with 40 nmol/L DiOC6, for 15 min, at 37 °C. After centrifugation, cells were washed, then suspended in 500 µL of PBS and analyzed by FACS with at least 1 × 10^4^ cells for each sample.

#### 4.4.3. Measurement of Intracellular Reactive Oxygen Species (ROS)

ROS level was evaluated by changes in fluorescence resulting from the intracellular oxidation of 10 μM (final concentration) dichloro-dihydro-fluorescein diacetate (DCFDA, Merck, Milan, Italy), added to the cell medium 30 min before the end of treatment. After trypsinization, cells were centrifuged, collected, washed and suspended in PBS, followed by FACS analysis. At least 1 × 10^4^ cells per sample were analyzed.

#### 4.4.4. Analysis of Cell Cycle

Cell cycle was analyzed by FACS. After treatment, cells were harvested by centrifugation, washed with PBS and incubated for 30 min, at room temperature in the dark in a PBS solution containing Triton X100 (0.1%, *v*/*v*), 20 μg/mL PI (both of them form Merck, Milan, Italy) and 200 μg/mL RNase (Thermo Fisher, Milan, Italy). Samples of at least 1 × 10^4^ cells were analyzed for each sample.

### 4.5. Western Blotting

After treatment, cells were rinsed with cold PBS, pH 7.0, and treated with cell lysis buffer (0.5% sodium deoxycholate, 150 mM NaCl, 0.1% SDS, 1% NP-40, 50 mM Tris-HCl, pH 7.4, 20 mM NaF, 50 mM glycerophosphate, 20 mM EGTA, 0.5 mM PMSF, 1 mM DTT, and 1 mM Na3VO4) containing phosphatase- and protease-inhibitors. The protein samples were withdrawn, transferred into a microtube, and vigorously vortexed for 15 s every 10 min, for a total of 30 min. The protein samples were centrifuged at 12,000 rpm, at 4 °C, for 30 min, and supernatants were collected and stored at −80 °C until use. Protein concentration was determined by Bradford reagent. Protein samples (80 μg/lane) were subjected to SDS-PAGE and transferred to nitrocellulose membrane as previously reported [45]. The immunoblot was incubated overnight at 4 °C with blocking solution (5% skim milk), followed by incubation with anti-PARP-1 monoclonal antibody (diluted 1/200, clone D-1, Cat No. SC-365315, Santa Cruz Biotechnology, Santa Cruz, CA, USA), anti-Bax monoclonal antibody (diluted 1/200, clone YTH6A7, Cat No. SC-80658, Santa Cruz Biotechnology), anti-Bcl-2 (diluted 1/200, clone C-2, Cat No. SC-7382, Santa Cruz Biotechnology) or anti-caspase 3 (diluted 1/200, clone 31A1067, Cat No. SC-56053, Santa Cruz Biotechnology), for 1 h at room temperature. Blots were washed two times with Tris-buffered saline/Tween 20 (TBST) and incubated with a 1:2000 dilution of horseradish peroxidase (HRP)-conjugated anti-IgG antibody (Dako Denmark, Glostrup, Denmark), for 1 h at room temperature. Blots were again washed five times with TBST and then developed by enhanced chemiluminescence (Amersham Life Science, Arlington Heights, IL, USA). Immunoreactions were also performed using β-actin antibodies (clone C4 Cat No. SC-47778, Santa Cruz Biotechnology) as loading controls.

### 4.6. Statistics

The results are given as means and standard deviations. Unless otherwise stated, three independent observations were performed for each experiment thrice replicated. Calculations and graphs were obtained using the INSTAT-3 statistical software (GraphPad Software, Inc., San Diego, CA, USA) with a test for normality followed by ANOVA and Tukey’s correction for multiple comparisons. In all cases, significance was accepted if the null hypothesis was rejected at the *p* < 0.05 level.

## Figures and Tables

**Table 1:** Calculated IC_50_ values for the anti-proliferative activity of HME in various human colon cancer cell lines at different time-points.

Incubation Time (h)	HCT-116	Caco-2	HT-29
	IC_50_ (mg Manna Equivalent/mL)		
24	10.73 ± 1.22	8.51 ± 0.77	28.92 ± 1.99
48	6.38 ± 0.51	5.88 ± 0.43	18.68 ± 1.54
72	4.25 ± 0.31	3.84 ± 0.29	13.07 ± 1.12

Values are the mean ± SD of four separate experiments in triplicate.
